# Supplementary material for: An Integrative Framework Identifies Cooperative Targeting of Host Pathways by Tick Salivary miRNAs
Source: Comput Struct Biotechnol J. 2026 May 15;35(1):0106. doi: 10.34133/csbj.0106 (PMC13176609; doi:10.34133/csbj.0106)
Supplement: Supplementary 1 — Figs. S1 to S3 Supplementary Files 1 to 8 [file csbj.0106.f1.zip › Supplementary Figures.docx]

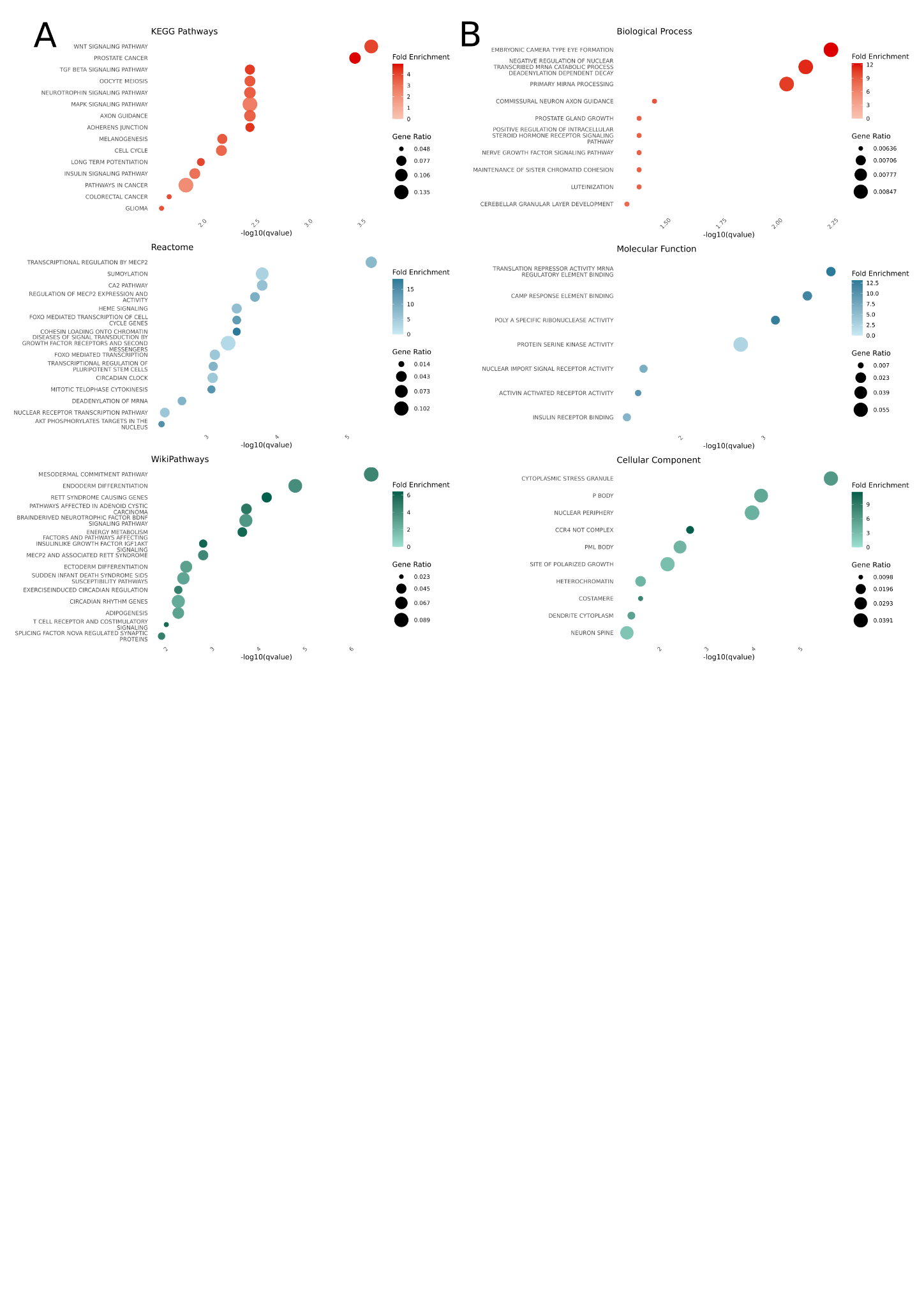


***Supplementary Figure 1. Functional enrichment analysis of genes with more than five conserved target sites for* Ixodes ricinus *microRNAs.***  *The functional enrichment of the specific genes was calculated using* ***A)*** *pathway databases: KEGG, Reactome and Wikipathways; and* ***B)*** *Ontology categories: Biological Process, Molecular Function and Cellular Component. The X-axis represents the statistical significance (-log10(qvalue)). The dots in the plot represent individual pathways, and their size is proportional to the Gene Ratio, which is the proportion of genes in the given pathway that have more than five conserved target sites for I. ricinus miRNAs, relative to the total number of genes in the pathway/ontology term. The intensity of color of each dot corresponds to the fold enrichment value, which reflects the percentage of genes with more than five conserved target sites belonging to a pathway, divided by the corresponding percentage of genes with conserved target sites (background) for the specific pathway.*


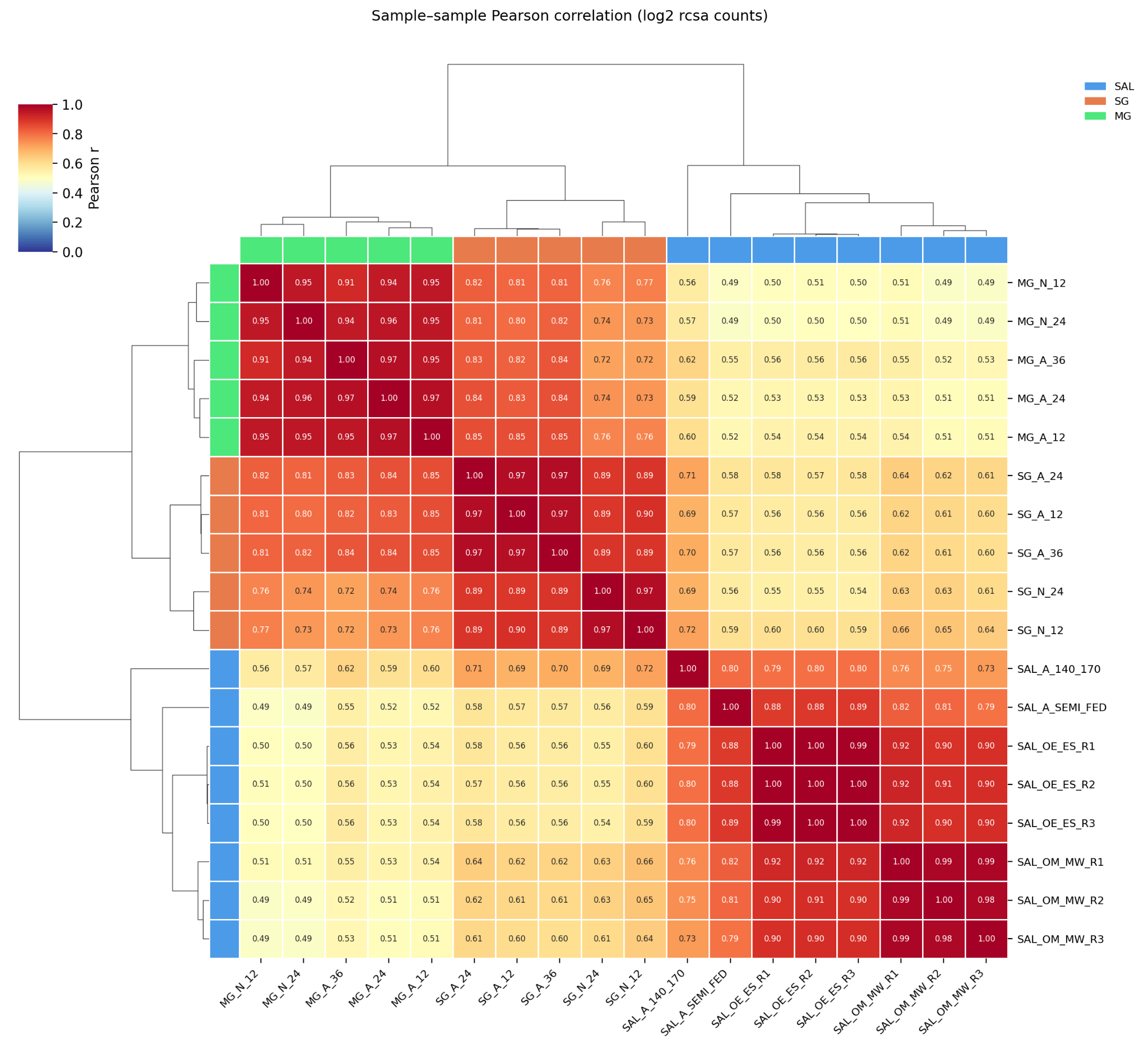


***Supplementary Figure 2. Sample-to-sample Pearson correlation of miRNA expression profiles across tick tissues.*** *Heatmap shows pairwise Pearson correlation coefficients (r) computed from log2-transformed rcsa-normalized miRNA counts for all 18 libraries. Rows and columns are ordered by hierarchical clustering (complete linkage). Color bars indicate tissue type: midgut (MG, green), salivary gland (SG, orange), and saliva (SAL, blue). Three distinct tissue clusters emerge: midgut libraries show the highest internal coherence (r = 0.91–1.00), followed by salivary gland libraries (r = 0.89–0.97), and saliva libraries (r = 0.79–1.00). Cross-tissue correlations are substantially lower, with saliva samples showing intermediate similarity to salivary glands (r = 0.56–0.72) and weaker correlation to midgut (r = 0.49–0.62), consistent with saliva representing a secreted subset of the salivary gland miRNA repertoire. Sample labels indicate tissue (MG/SG/SAL), species abbreviation, and feeding stage or replicate number.*


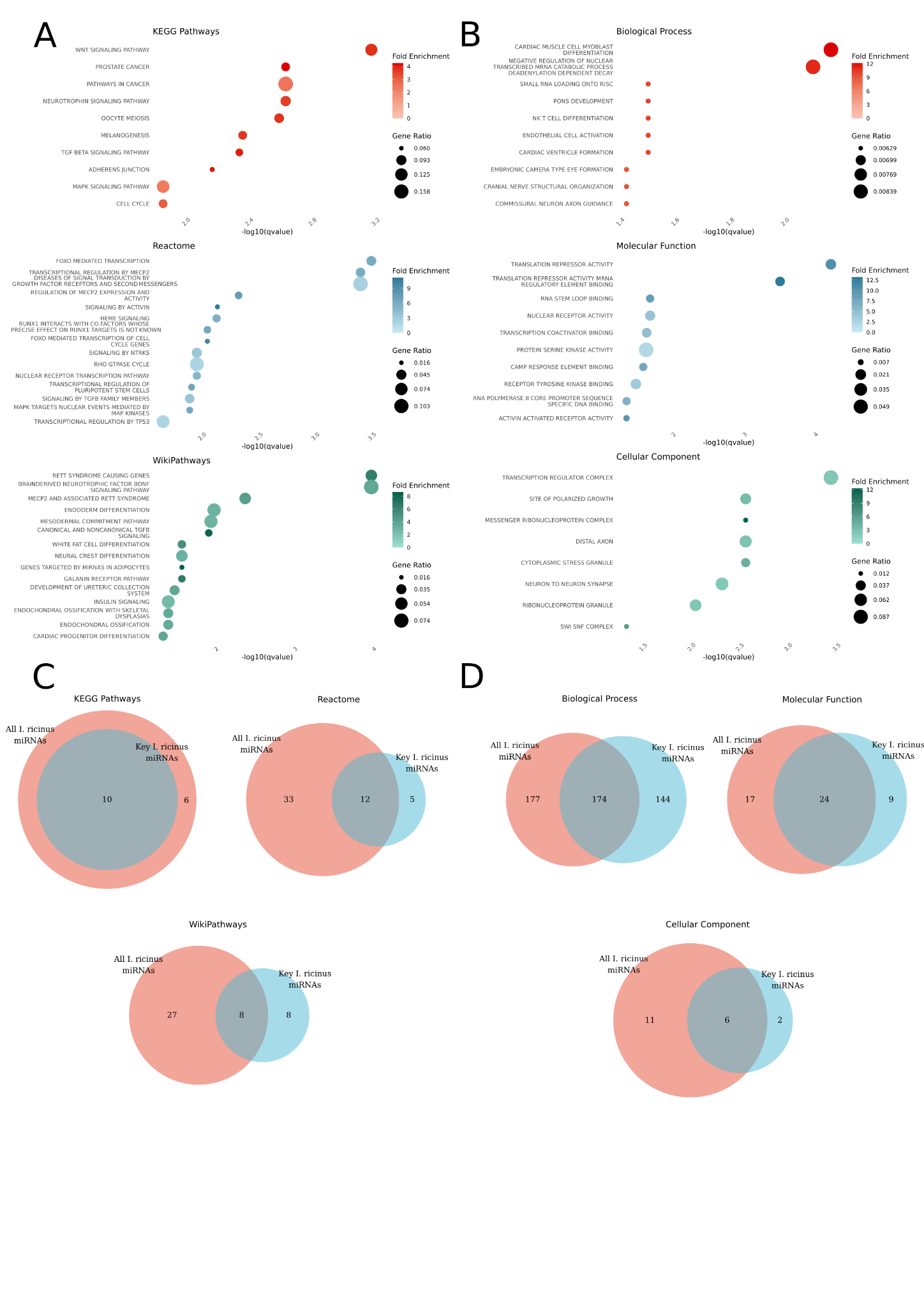


***Supplementary figure 3. Functional enrichment analysis of the 526 genes with more conserved target sites for core* Ixodes ricinus *salivary microRNAs.***  *The functional enrichment of the specific genes was calculated using* ***A)*** *pathway databases: KEGG, Reactome and Wikipathways; and* ***B)*** *Ontology categories: Biological Process, Molecular Function and Cellular Component. The X-axis represents the -log10(p-value), which reflects the statistical significance of the pathway enrichment. The dots in the plot represent individual pathways, and their size is proportional to the Gene Ratio, which is the proportion of genes in the given pathway targeted by core I. ricinus salivary miRNAs, relative to the total number of genes in the pathway. The intensity of color of each dot corresponds to the fold enrichment value, which reflects the percentage of genes with conserved target sites for the key miRNAs belonging to a pathway, divided by the corresponding percentage of genes with conserved target sites for all I. ricinus miRNAs (background) for the specific pathway.* ***C)*** *Comparison of pathway enrichment analysis for genes with conserved target sites between the full set and core I. ricinus salivary miRNAs. The analysis was performed for the three different pathway databases: KEGG pathways, Reactome pathways and Wikipathways.* ***D)*** *Comparison of ontology enrichment analysis for genes with conserved target sites between the full set and core I. ricinus salivary miRNAs. The analysis was performed for the three ontology categories: Biological Process, Molecular Function, and Cellular Component.*
