# Supplementary figures and images for: An Integrative Framework Identifies Cooperative Targeting of Host Pathways by Tick Salivary miRNAs
Source: Comput Struct Biotechnol J. 2026 May 15;35(1):0106. doi: 10.34133/csbj.0106 (PMC13176609; doi:10.34133/csbj.0106)

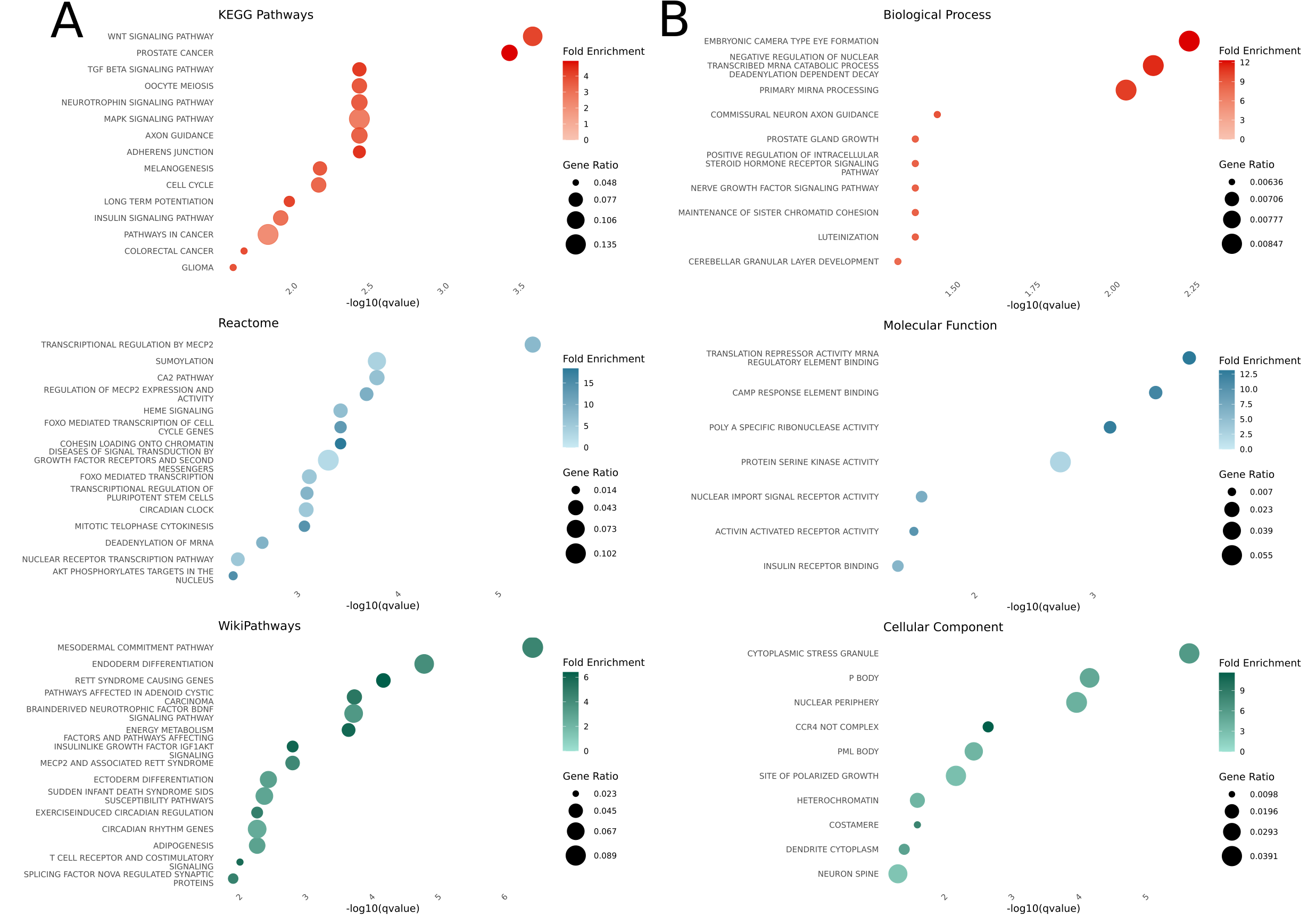

Supplement: Supplementary 1 — Figs. S1 to S3 Supplementary Files 1 to 8 [file csbj.0106.f1.zip › supplementary_figure_1.png]

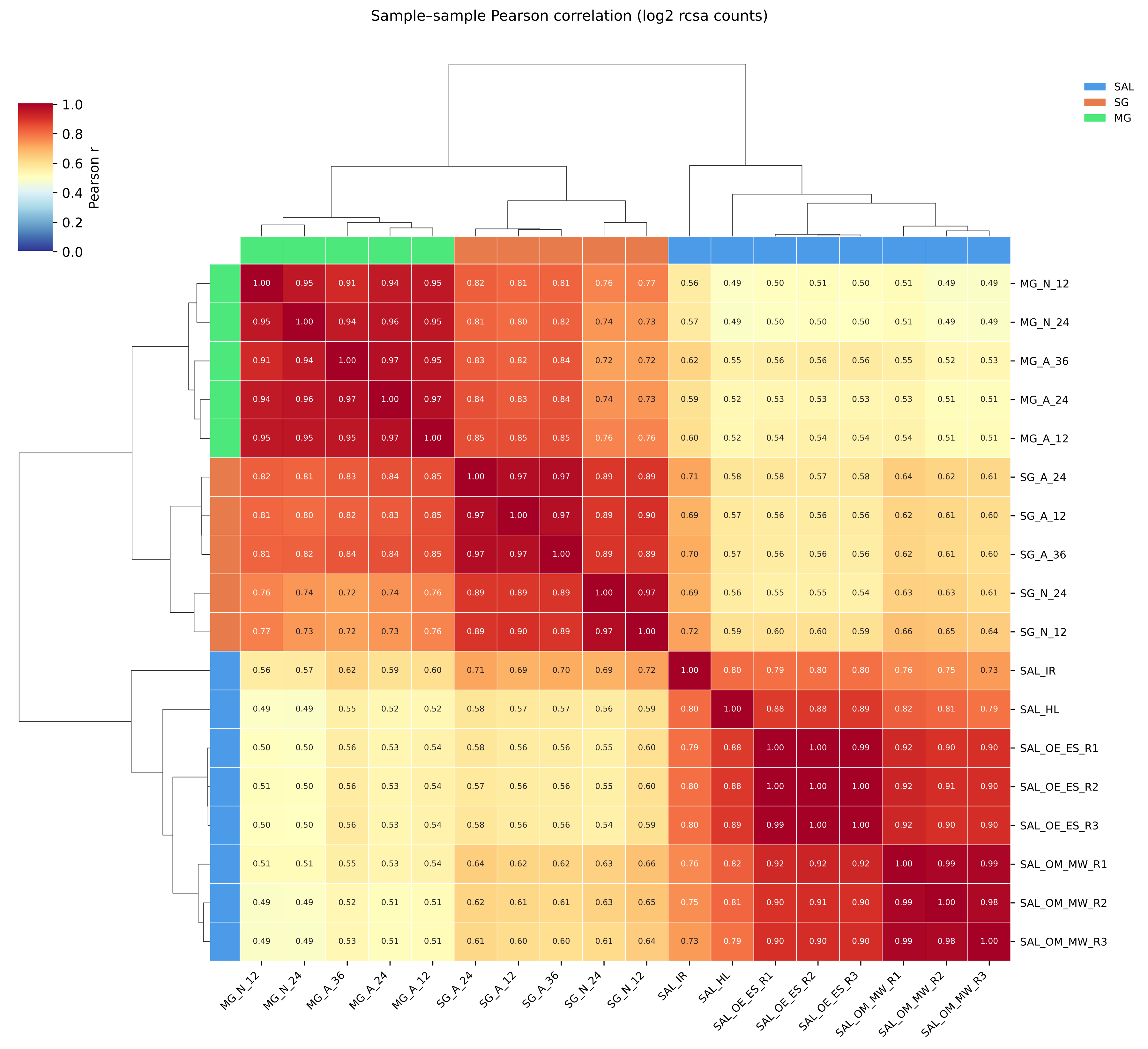

Supplement: Supplementary 1 — Figs. S1 to S3 Supplementary Files 1 to 8 [file csbj.0106.f1.zip › supplementary_figure_2.png]

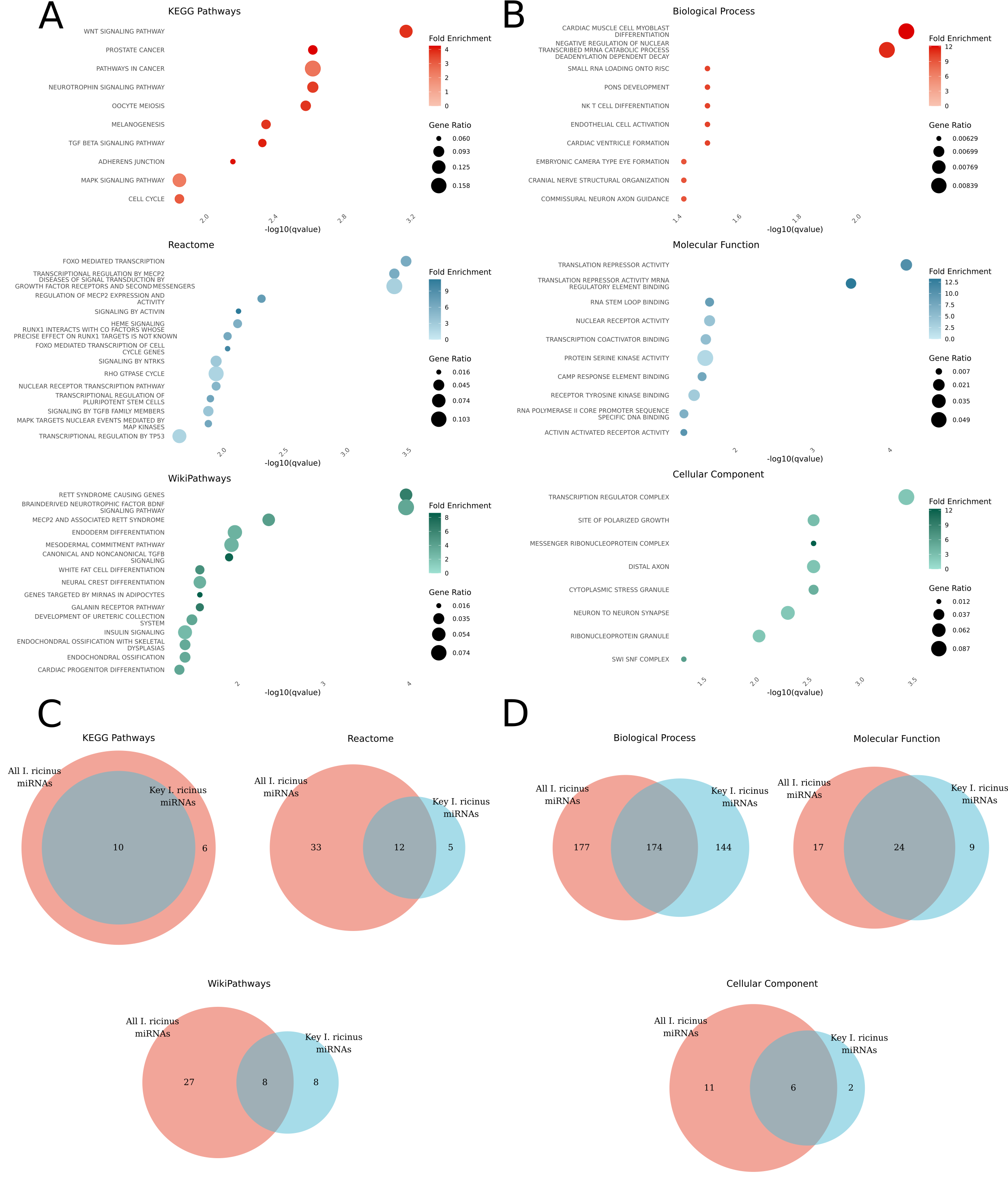

Supplement: Supplementary 1 — Figs. S1 to S3 Supplementary Files 1 to 8 [file csbj.0106.f1.zip › supplementary_figure_3.png]
